# Raman scattering enhancement of dielectric microspheres on silicon nitride film

**DOI:** 10.1038/s41598-022-09315-5

**Published:** 2022-03-29

**Authors:** Toshihiko Ogura

**Affiliations:** Health and Medical Research Institute, National Institute of Advanced Industrial Science and Technology (AIST), Central 6, Higashi 1-1-1, Tsukuba, Ibaraki 305-8566 Japan

**Keywords:** Micro-optics, Imaging and sensing, Microscopy, Raman spectroscopy

## Abstract

Circulating light in the total internal reflection within dielectric spheres or disks is called the whispering gallery mode (WGM), which by itself is highly sensitive to its surface and capable of detecting viruses and single atomic ions. The detection site of the sensors using WGM is created by the evanescent light from the circulating light inside spheres. Here we report anomalous Raman scattering enhancement in dielectric microspheres on a silicon nitride (SiN) film. This Raman enhancement occurs at the periphery of the spheres, and a similar ring of light was also observed under a fluorescence microscope. This is caused by the light circulating around the dielectric spheres as in the WGM. We observed anomalously enhanced Raman spectrum at the periphery of 3 μm diameter polystyrene (PS) microspheres on a SiN film using confocal laser Raman microscopy. The wavelength intensity of this enhanced Raman spectrum was accompanied by periodic changes due to interference. These features may lead to the development of high-sensitive sensors and optical devices.

## Introduction

Dielectric spheres and disks show peculiar properties such as WGM^1–8^, Mie scattering^9–12^ and surface enhanced Raman spectrum (SERS)^13–16^. These phenomena are generated by electric and magnetic dipole resonances inside dielectric micro- or nanospheres, disks and cylinders by light irradiation^17–19^. The incident light on a dielectric microsphere circulates at the internal surface of the sphere with total reflection^17–19^. At this time, the incident light is drastically amplified and exhibits a high-quality factor WGM^5–7^. The surface of the WGM devices generates evanescent light due to the internally orbiting light and functions as an extremely sensitive sensor for viruses^4^, proteins^2^ and single atomic ions^3^. In addition, WGM of the microspheres and disks can be applied to sensors^1–4^, resonators^5–8^, laser^20–24^, quantum and nonlinear optics^25–27^.

Recently, we have developed a new imaging technology, scanning electron-assisted dielectric microscopy (SE-ADM)^28,29^ based on scanning electron microscope, which enables the observation of various biological specimens in aqueous media at 8 nm spatial resolution^29^. In this method, the biological samples are enclosed in a liquid holder composed of tungsten-coated SiN film and are not directly exposed to electron beam, thus minimizing electron radiation damage^30,31^. As a next step, we attempted to observe test samples using a confocal laser Raman microscope using this sample holder with a 50 nm thick SiN film. Using 3 μm PS spheres, we detected anomalous Raman spectrum enhancements around the sphere.

Here, we have investigated the conditions under which this anomalous Raman scattering enhancement occurs, varying the diameter of the PS spheres and the thickness of the SiN film. Furthermore, we discuss whether this phenomenon is related to WGM^32^, photonic nanojet^33–35^, or Fano resonance^36,37^ since this abnormal Raman shift covers the surface of the dielectric sphere. This orbiting light like a WGM was induced when the sphere was attached to a SiN film but not detected on other substrates such as a slide glass or a SiN film with Si frame. Moreover, the orbiting light like a WGM strongly influenced by the thickness of SiN film and the sphere diameter. This circulating light can be observed under a confocal laser Raman microscopy and conventional fluorescence microscopy. This orbiting light on the microsphere is extremely sensitive to the surface condition of the spheres and the contact site of the SiN film, and is expected to be used for highly sensitive compact sensors, antennae and optical quantum elements.

## Results

### Anomalous Raman spectrum of dielectric microspheres on a SiN film

**Figure 1:**
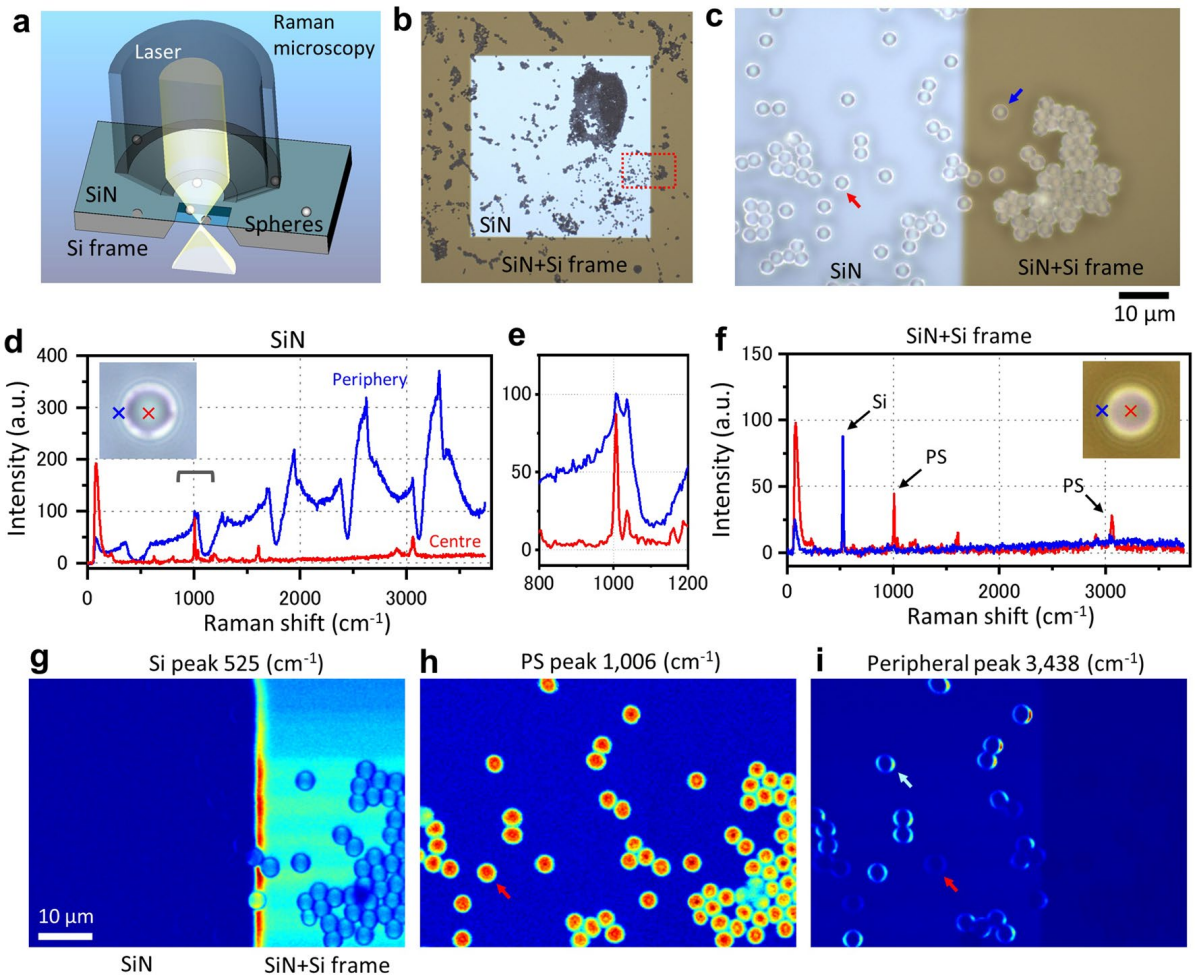
Raman spectrum of PS microspheres on SiN film. (**a**) Schematic of the setup used to measure the Raman spectrum of 3 μm diameter PS spheres on a SiN film of 50 nm thickness supported by a Si frame. (**b**) OM image of the spheres on a 0.4 × 0.4 mm square SiN film. (**c**) Higher magnification OM image (×1000) of the boundary region between the SiN film and the Si frame (dashed red rectangle in **b**). (**d**) Raman spectra of the sphere centre (red line) and left periphery (blue line) on the SiN film indicated by a red arrow in (**c**). The sphere centre exhibited a PS spectrum, while the periphery showed a very high intensity spectrum with periodicity. (**e**) Raman spectrum of (**d**) focusing on a range of 800–1200 cm^−1^. At the sphere centre, the PS Raman peak of 1006 cm^−1^ was clear (red line), while the PS peak of the sphere periphery was very low (blue line). (**f**) Raman spectra of the sphere centre (red line) and left periphery (blue line) on the SiN film contacted by a Si frame indicated by a blue arrow in (**c**). Under this condition, similar PS and Si peaks were observed in both Raman spectra. (**g**) Raman image of Si peak (525 cm^−1^) in (**c**). The Si frame on the right side was clearly observed. (**h**) With the Raman image of PS peak (1006 cm^−1^), all spheres in the scanned area were clearly observed. (**i**) With the Raman image of 3438 cm^−1^, the spheres on the SiN film showed a ring shape. Scale bars, 10 μm in (**c**) and (**g**).

PS microspheres attached on a SiN film were observed under a confocal laser Raman microscope (Fig. 1a). The 3 μm diameter spheres were dispersed on a SiN film of 50 nm thickness supported by a Si frame with a square window; the SiN film over the window was not directly contacted by the Si frame (Fig. 1b,c). We compared Raman spectra of PS spheres on the SiN film with and without direct contact of the Si frame (Fig. 1d–f). For this observation, a confocal laser Raman microscope was used, where two pinhole apertures, one in front of the laser and one in front of the detector, allowed the light to be detected from the focus position only^38–40^. Therefore, it was possible to remove scattered light and stray light deviating from the focal position and perform three-dimensional measurement of the sample^39,40^. At the centre of the sphere on the SiN film without contact with the Si frame, the Raman spectrum showed a PS peak of 1006 cm^−1^ (Fig. 1d red line). Notably, we observed anomalously enhanced Raman spectra with a periodic amplitude change at the periphery of spheres (Fig. 1d blue line). Furthermore, the PS peak at 1006 cm^−1^ was very small, demonstrating that the laser did not irradiate the inside of the spheres directly (Fig. 1e). Moreover, this result suggests that the incident light does not circulate inside the spheres. For PS spheres attached on the SiN film contacted by a Si frame, Raman peaks of Si at 525 cm^−1^ and of PS at 1006 cm^−1^ were observed at the centre and periphery, but without any periodic anomalous Raman spectra (Fig. 1f).

For further analysis of this observation, we performed a two-dimensional (2D) Raman spectrum scanning of the image in Fig. 1c, and Raman images were calculated using the Raman peaks of Si (525 cm^−1^), PS (1006 cm^−1^) and the anomalous Raman spectrum (3438 cm^−1^) (Fig. 1g–i). In the Si peak image of Fig. 1g, the Si frame on the right side showed high-intensity region. At the edge of the Si frame of the image centre, the Si spectrum was enhanced, which is an effect by conventional SERS^13,19^. At the PS peak of 1006 cm^−1^, as expected, all the spheres in the scanned area showed a spherical shape similar to the optical microscopy (OM) images (Fig. 1h). Interestingly, with the peak of the anomalous Raman spectrum at 3438 cm^−1^, the ring-like shapes were observed only for the spheres on the SiN film without direct contact with the Si frame, but not at all for those with contact (Fig. 1i). Furthermore, anomalous Raman spectra were not observed for spheres on slide glass (Supplementary Fig. 1).

**Figure 2:**
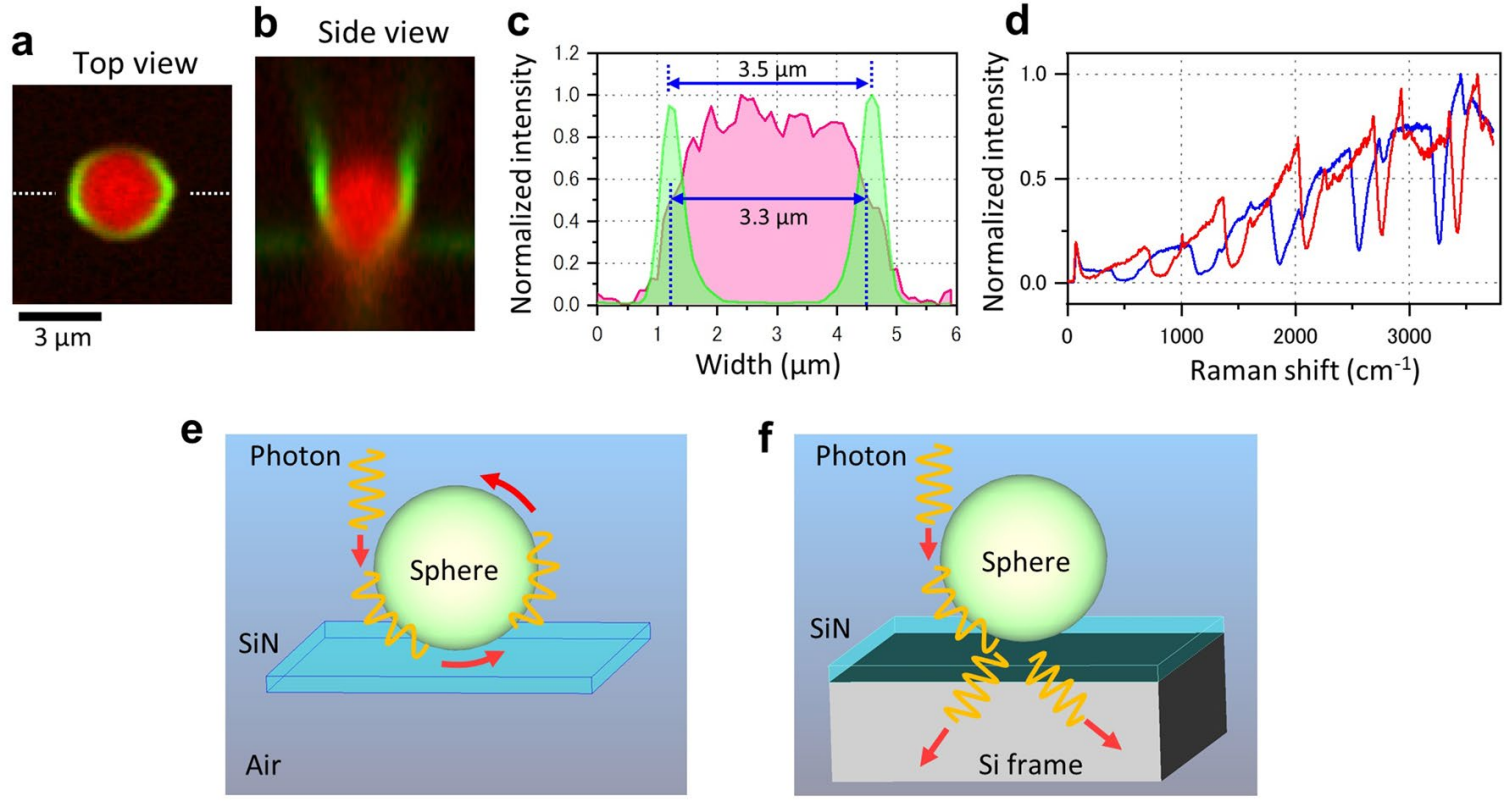
Raman spectra of the sphere circumference on SiN film. (**a**,**b**) Top and side views of merged Raman images of the PS peak (1006 cm^−1^, red) and anomalous peak of 3438 cm^−1^ (green) with a 3 μm diameter sphere on a 50 nm thick SiN film without contact with a Si frame. The PS sphere is seen to be covered with a ring-shaped anomalous Raman peak. (**c**) Comparison of the line plots of PS peak (1006 cm^−1^, red) and 3438 cm^−1^ (green) at the sphere centre in (**a**). The Raman intensities are normalized individually. The distance between the two half maxima of the PS peak is 3.3 μm and the distance between the peaks at the periphery of the 3438 cm^−1^ is 3.5 μm. (**d**) Comparison of the Raman spectrum at two sphere circumferences in Fig. 1i indicated by red and blue arrows. Both spectra were of 4 cycles but the phases were different. (**e**) In the spheres on a SiN film, the light continues to circulate without being affected by the film. (**f**) In the spheres on a SiN film directly on a Si frame, the orbiting light is scattered and lost due to the influence of the Si frame. Scale bar, 3 μm in (**a**).

Next, we analysed the Raman images of the spheres on the SiN film without direct contact with the Si frame (Fig. 2 and Supplementary Fig. 2). The Raman spectrum of the sphere periphery on the SiN film showed a very large signal with periodic intensity changes (Supplementary Fig. 2a–c). The top-view 2D Raman images of the microsphere showed a dome-shaped PS peak at 1006 cm^−1^ and a ring-shaped anomalous Raman spectrum peak (Supplementary Fig. 2d top). From the side, the Raman images of the PS peak and reflecting peak of 72 cm^−1^ showed a spherical shape similar to the top view image, and the anomalous Raman image was clearest at the equatorial point of the spheres (Supplementary Fig. 2d bottom). For the spheres on the SiN film with Si frame contact, the anomalous Raman spectrum was hardly visible and no ring structure was observed (Supplementary Fig. 3). In the superimposed image of the PS peak and the anomalous Raman peak, the anomalous Raman spectrum was found to cover the surface of the sphere (Fig. 2a,b). Along a line drawn through the sphere, the PS signal of 1006 cm^−1^ showed a dome-like shape, while the anomalous Raman spectrum peak of 3438 cm^−1^ showed sharp peaks on both sides of the sphere (Fig. 2c). The width at half maximum of the PS signal was 3.3 μm, almost equal to the sphere diameter of 3 μm. The width between the two anomalous Raman spectrum peaks was slightly larger, 3.5 μm, indicating that this Raman spectrum existed only on the surface of the spheres. This result indicates that the incident light covers the circumference of the PS sphere. Therefore, the incident light is considered to be orbiting outside the perimeter of the sphere. Furthermore, the periodicity and phase of the anomalous Raman spectra differed from sphere to sphere (Fig. 2d). These differences may be due to the diameter of the spheres.

Based on these experimental results, we propose that the anomalous Raman spectrum was generated by irradiated light orbiting around the surface of dielectric spheres (Fig. 2e). When light is incident on a sphere, a part of the waveform on one side of the photon enters the sphere and bends its path along the curved surface (Fig. 2e). The refractive index of the PS sphere is approximately 1.6^41,42^, higher than that of air; thus, the light bends along the surface of the sphere upon reaching the surface. The light orbiting around the sphere surface travels towards the SiN film at the bottom, where it should continue its orbit without being scattered and then returns to the top (Fig. 2e). For spheres on glass or SiN film with Si frame contact, the orbiting light is scattered by the glass or Si frame, but does not return to the top of the sphere (Fig. 2f). When two or three spheres on a SiN film are in close proximity, the anomalous Raman spectra show attenuation areas (Supplementary Fig. 4a–j). These attenuation areas are located on the line connecting the sphere centres and the sphere contact position (Supplementary Fig. 4d and i). This is because the incident light circulates around the surface of the spheres and is attenuated at the contact position of the spheres on the opposite side (Supplementary Fig. 4k). This result also strongly supports the phenomenon of light circling around the spheres.

### SiN thickness dependence of anomalous Raman spectrum

**Figure 3:**
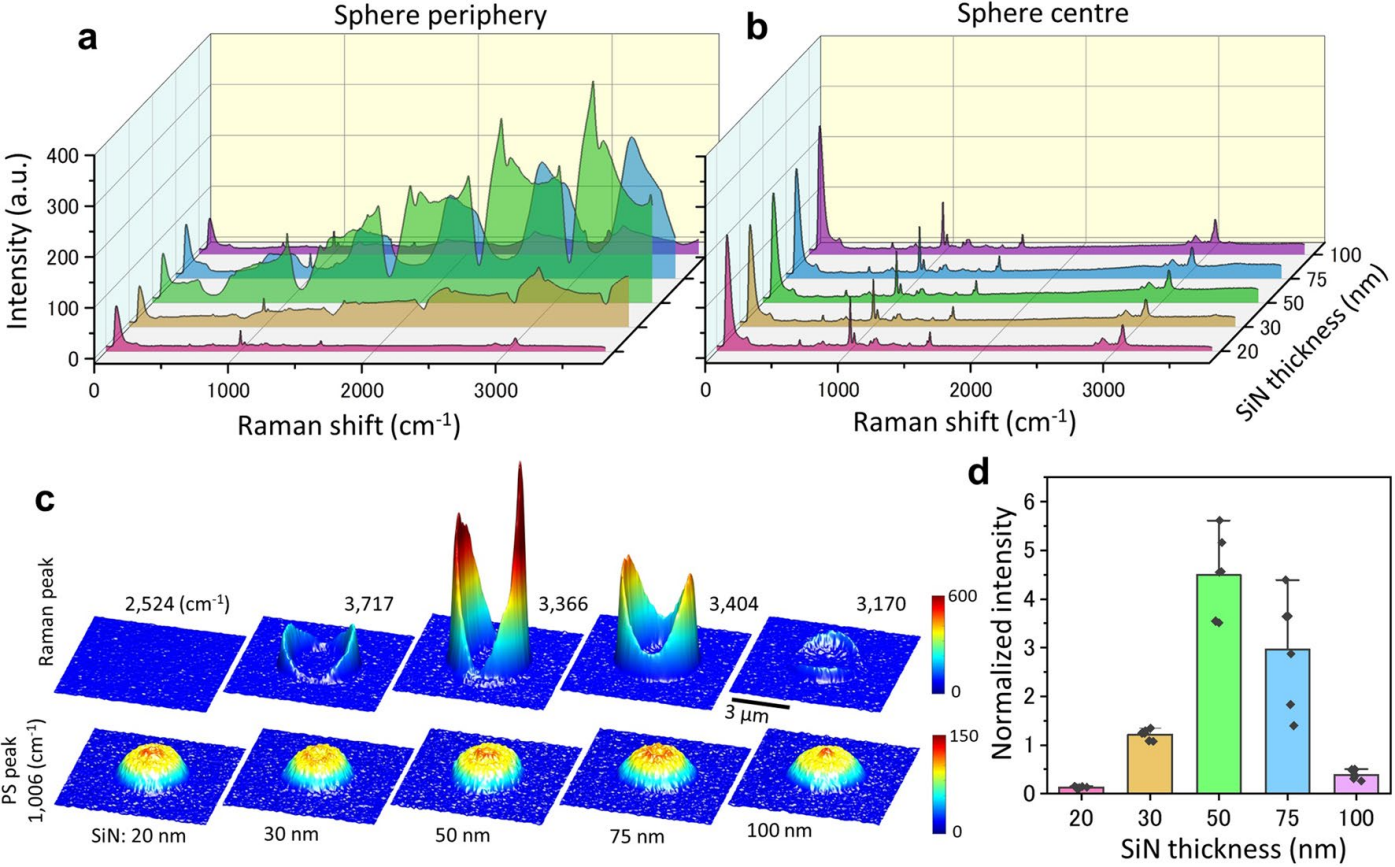
Relationship between SiN thickness and the intensity of the sphere’s circumferential light. (**a**) The average Raman spectra of the circumference of the 3 μm diameter PS spheres with a SiN thickness of 20–100 nm. The circumferential light was most intense with the SiN film of 50 nm thickness but hardly detectable with a SiN film of 20 or 100 nm thickness. (**b**) Average Raman spectrum at the sphere centre. The same PS spectra were seen for all the SiN film thicknesses. (**c**) Pseudo-3D colour maps of the PS peaks and the anomalous Raman peaks for SiN thicknesses of 20–100 nm. The maps of the PS peak showed a similar dome-like shape for all SiN thicknesses (bottom row). With the anomalous Raman peaks, the ring shape around the spheres was clearly observed at the SiN thicknesses of 50 and 75 nm (top row). (**d**) Relationship between the anomalous Raman peaks and SiN thickness. Six spheres were measured at each SiN thickness and the ratios of the maximum anomalous Raman intensity to that of the PS peak (1006 cm^−1^) at the sphere centre were calculated and normalised. The normalized intensity of the Raman spectrum was obtained by normalizing the maximum Raman intensity around the PS sphere at the PS peak (1006 cm^−1^) at the centre of the sphere. The mean values of normalized intensity from 20 to 100 nm thickness are 0.13, 1.22, 4.49, 2.97 and 0.38 respectively. Standard deviations are 0.018, 0.109, 0.846, 1.158 and 0.097. Anomalous Raman intensity had a maximum at 50 nm SiN thickness but was reduced when the SiN thickness was increased or decreased. Scale bar, 3 μm in (**c**).

Light orbiting on the sphere can continue its orbit without being scattered on the SiN film. To verify this mechanism, we measured and compared anomalous Raman spectra of spheres on SiN films of different thickness (Fig. 3). If the light orbiting the surface of the sphere simply transmits through the SiN film, the intensity of light transmission will increase with a thinner SiN film. Therefore, the intensity of the anomalous Raman spectrum should increase when the thinner SiN film, and decrease with increasing SiN thickness. Figure 3a,b show the Raman spectra at the centre and periphery of 3 μm spheres on SiN films of 20 to 100 nm thickness. The anomalous Raman spectrum around the sphere was the largest for the 50 nm thickness, and was hardly detected for the SiN films of 20 or 100 nm thickness (Fig. 3a). At the sphere centre, only the Raman spectra of PS signal were seen for any film thickness (Fig. 3b). In the 3D pseudo-colour map of the spheres, the anomalous Raman spectrum was clearly seen surrounding the sphere surface on 50 nm thick SiN films (Fig. 3c). However, the anomalous Raman spectrum decreased upon varying the thickness from 50 nm (Fig. 3c,d). These results suggest that the circumferential light does not simply pass through the SiN film but that there must be a mechanism that assists the light circulating most efficiently at 50 nm thickness.

**Figure 4:**
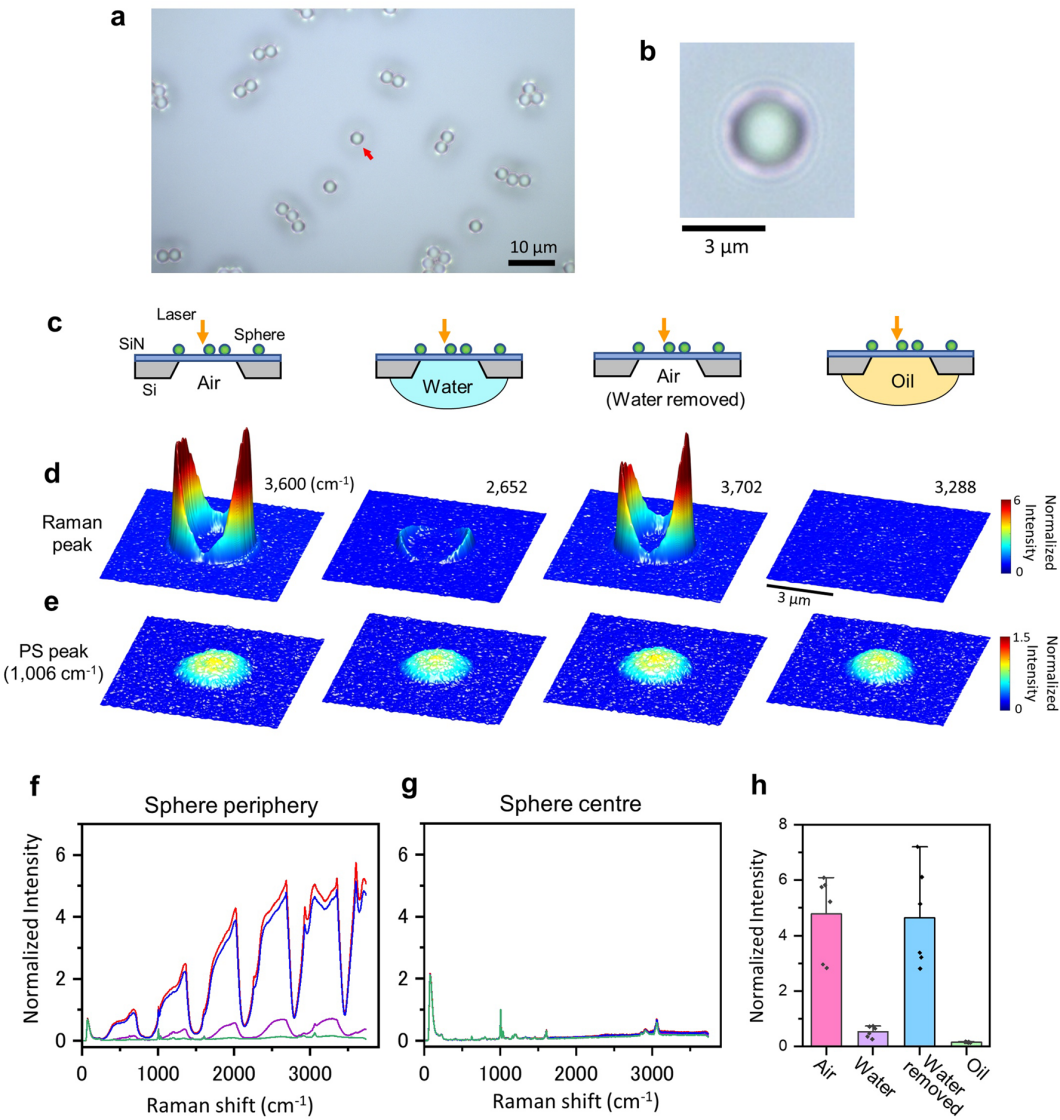
Raman spectrum of PS microspheres on the SiN film with water or oil underneath the film instead of air. (**a**) An OM image of 3 μm diameter PS spheres on the 50 nm thick SiN film. (**b**) Magnified sphere image indicated by a red arrow in (**a**). (**c**) Schematic of the measurement of Raman spectrum of 3 μm diameter PS spheres on a SiN film with water or immersion oil underneath the film. (**d**) Pseudo-3D colour maps of the anomalous Raman peaks on a SiN film with water or oil underneath the film. (**e**) Pseudo-3D colour maps of PS Raman peaks on a SiN film with water or oil underneath of the film. The sphere structures were observed in all the cases. (**f**) Raman spectra of sphere peripheries normalized by PS peaks of 1006 cm^−1^ of the sphere centre on a SiN film with air, water or oil underneath the film. The anomalous periodic Raman spectra are seen with the air underneath the film (red, with air in (**c**) and (**d**) left and blue, with removal of water in (**c**) and (**d** the third from the left). With water underneath the film, the anomalous Raman spectra decreased (purple, with water, in (**c**) and (**d**) the second from the left). With oil underneath the film, the Raman spectra indicated only the PS spectrum (green, with oil, in (**c**) and (**d**) right). (**g**) Normalized Raman spectra of sphere centre by PS peaks of 1006 cm^−1^ at the sphere centre on a SiN film with air, water or oil underneath the film. The Raman spectra under any condition show only the PS spectrum. (**h**) Relationship between the anomalous Raman peaks around the spheres and the SiN film with air, water or oil underneath the film. Six sphere samples were measured under each condition. The mean value of normalized intensity in each condition is 4.78 (air), 0.53 (water), 4.64 (water removed) and 0.15 (oil). Standard deviations are 1.48, 0.20, 1.79 and 0.02. Scale bars, 10 μm in (**a**), 3 μm in (**b**) and (**d**).

In order to investigate the light at the bottom of the 50 nm thick SiN film, the Raman spectra of the spheres were measured with either water or immersion oil underneath the SiN film in place of air (Fig. 4). The refractive index of the SiN is approximately 2.0^43,44^. The pure water and immersion oil used here transmit light well, but their refractive indices of 1.33 and 1.52 are higher than that of air at 1.0. The Raman spectral intensity around the sphere was significantly attenuated when water was filled underneath the SiN film (Fig. 4a–e). The spectral intensity was regained when the water was removed and replaced with air. When oil was filled underneath the SiN film, the Raman spectra around the spheres disappeared completely (Fig. 4f–h, Supplementary Fig. 5).

### Sphere diameter and anomalous Raman spectrum

**Figure 5:**
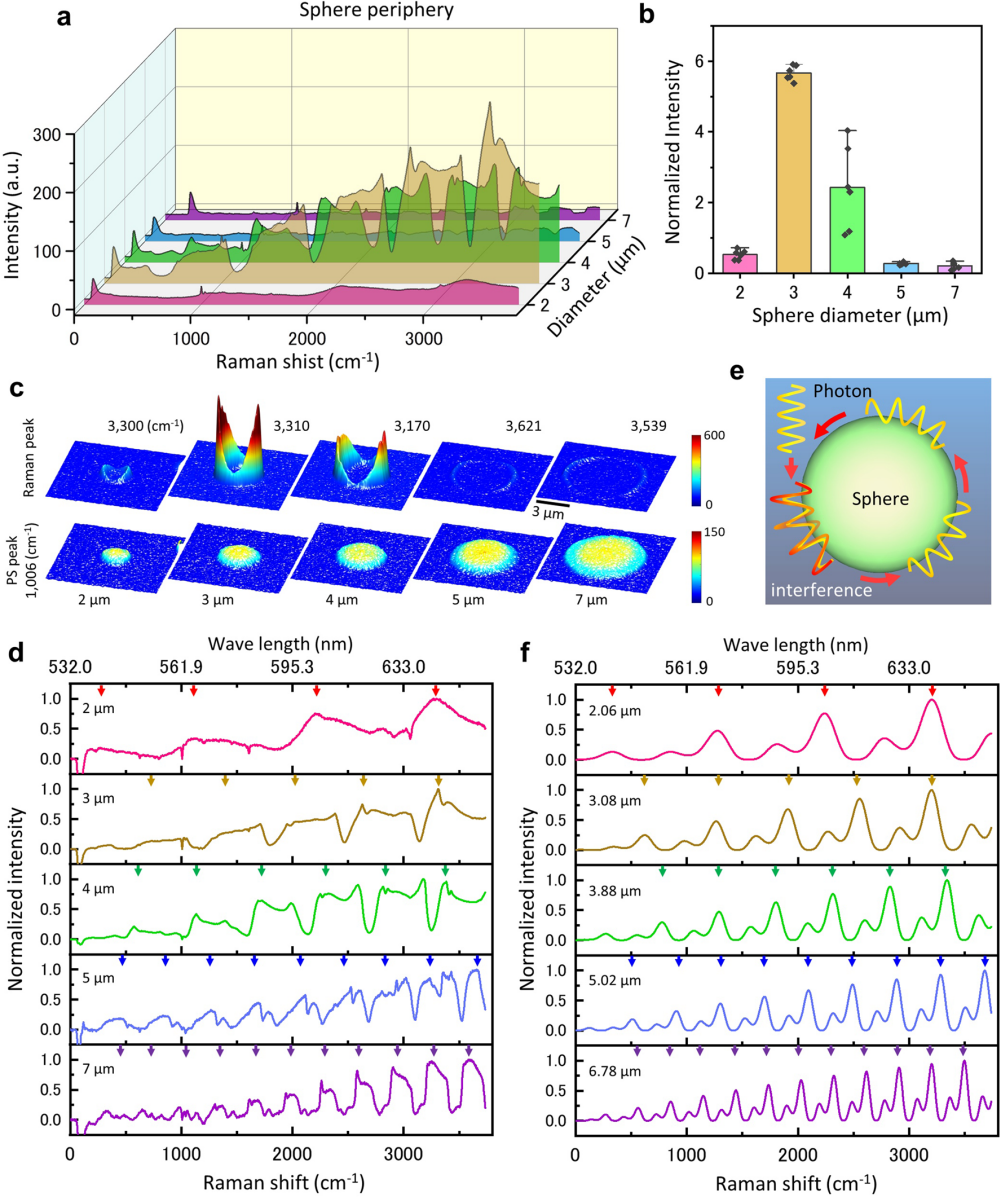
Sphere size and circulating light intensity. (**a**) Averaged Raman spectra of the circumference of PS spheres of 2–7 μm diameter on a 50 nm thick SiN film. (**b**) Relationship between the anomalous Raman peaks and sphere diameter. Six spheres were measured at each diameter and the ratio of the maximal anomalous Raman intensity to the PS peak (1006 cm^−1^) at the sphere centre was calculated and normalised. The mean values of normalized intensity from 2  to 7 μm diameter are 0.527, 5.66, 2.43, 0.274 and 0.207 respectively. Standard deviations are 0.137, 0.213, 1.196, 0.032 and 0.099. The anomalous Raman peak intensity was the highest at the sphere diameter of 3 μm but was very low at 2 μm and 5–7 μm. (**c**) Pseudo-3D colour maps of the PS peak and the circumferential peak for sphere diameter of 2–7 μm. The maps of the PS peak show similar dome-like shape for all diameters (bottom row). With the anomalous Raman peak, the ring shape around the spheres was clearly observed at the SiN thickness of 3 and 4 μm (top row). (**d**) Normalized Raman spectrum of the sphere circumference with a diameter of 2–7 μm. The arrows in the figure indicate the peaks of each spectral periodicity. With the sphere diameter increment from 2 to 7 μm, the periodicity of the Raman spectrum increases from 4 to 11. (**e**) Schematic of the interference produced by light circulating around the sphere. A photon travelling around the periphery of the sphere interferes with other photons while they travel around the sphere. (**f**) Raman spectrum of sphere periphery by the numerical model of Eq. (1). The sphere diameters of the model were set to the values where the period and phase were in close correspondence with the experimental results (left side diameters in each plot). Scale bar, 3 μm in (**c**).

By examining the sphere diameter with the maximum intensity of the anomalous Raman spectrum, it is possible to compare the conditions for the generation of the circumferential light with the mathematical model. In addition, by investigating the cycle of the anomalous Raman spectrum intensity and the sphere diameter, the generation mechanism can be determined. We obtained the Raman spectra of spheres with diameters of 2–7 μm on a 50 nm thick SiN film (Fig. 5a). With a sphere diameter of 3 and 4 μm, the anomalous Raman spectrum around the sphere was significantly increased (Fig. 5a,b). In contrast, with a sphere diameter of 2 μm and 5 μm or more, the anomalous Raman spectrum was very weak. In the 3D pseudo-colour map of the spheres, the anomalous Raman spectrum was clearly seen surrounding the sphere with the diameter of 3 and 4 μm (Fig. 5c). For other diameters, the spectra were very weak. Figure 5d shows the normalised Raman spectrum of the sphere periphery after subtracting the PS Raman spectrum at the sphere centre. For a diameter of 2 μm, the Raman spectrum changed with a slow cycle, approximately 4 cycles (Fig. 5d top). The periodicity of this spectrum gradually increased with an increasing sphere diameter, reaching 11 cycles with a diameter of 7 μm. These gentle and periodic changes of the Raman spectrum are presumably due to interference^45^. The orbiting photon around the spheres is expected to be interfered with in the process of repeated orbiting on the sphere surface (Fig. 5e). Model calculations show that the periodicity of this interference increases with increasing diameter of the sphere (Fig. 5f).

The relationship between optical power intensity *P*(*λ*) and interference is expressed by the following equation (see details under “Materials and methods”).

1 $$P\left(\lambda \right)=R\left(\lambda \right){\left[\frac{1}{n}\sum \limits_{m=1}^{n}\mathrm{sin}\left(2\pi \frac{\pi {\mathit{mD}}_{s}{\eta }_{s}}{\lambda }\right)+1\right]}^{2}$$

Here, *λ* is wavelength, *R*(*λ*) is amplitude coefficient relative to wavelength, *D*_*s*_ is the sphere diameter, *η*_*s*_ is refractive index of PS of 1.6^41,42^, and *n* is the number of light orbits and is set to 2. Figure 5f shows the optical power of orbiting light calculated by Eq. (1) at wavelengths between 532 and 640 nm and for sphere diameters between 2 and 7 μm. For a diameter of 2 μm, the change in light intensity is about 4 cycles in the numerical model (Fig. 5f top). In the case of diameters of 3 to 7 μm, the periodicity of the intensity change was 5 to 11 (Fig. 5f), which agreed with the experimental results (Fig. 5d). However, the waveform on the positive side of the model rises slightly more sharply than the experimental results.

### Comparison of fluorescence images of spheres attached to the SiN film and glass

**Figure 6:**
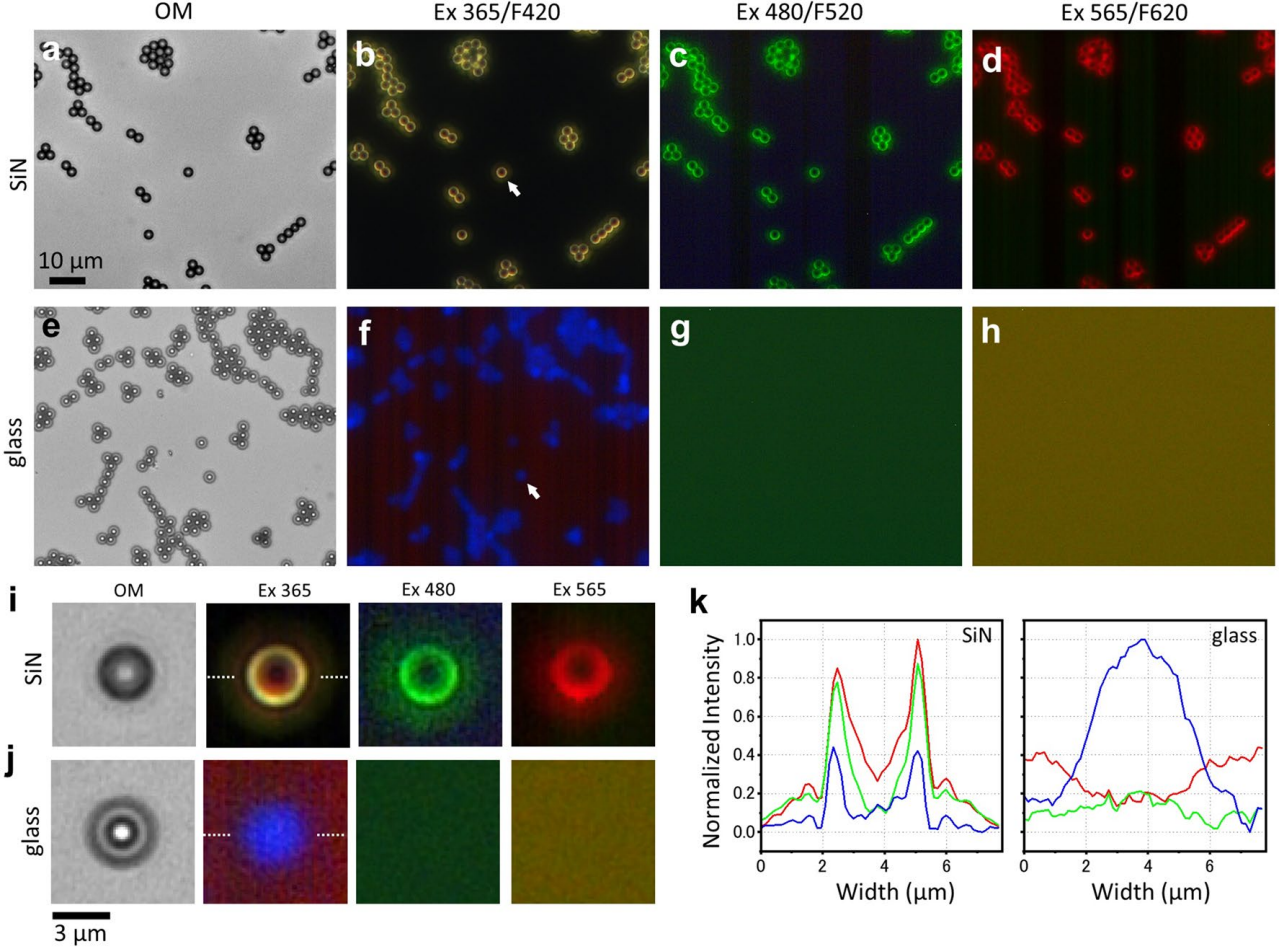
Comparison of PS spheres attached to glass and SiN film by fluorescence microscopy. (**a**) OM image of the 3 μm diameter PS spheres on a 50 nm thick SiN film. (**b**–**d**) Images of spheres attached to the SiN film using three sets of fluorescence filters with excitation at 365 nm and fluorescence at 420 nm (Ex365/F420), excitation at 480 nm and fluorescence at 520 nm (Ex480/F520) and excitation at 565 nm and fluorescence at 620 nm (Ex565/F620). The PS spheres attached to the SiN film were clearly observed with all three sets of filters. (**e**) OM image of the 3 μm diameter spheres attached to the slide glass. (**f**–**h**) Images of spheres attached to the slide glass using three sets of fluorescence filters with excitation at Ex365/F420, Ex480/F520 and Ex565/F520. The spheres attached to the glass showed very weak autofluorescence with a set of filters of Ex365/F420, and the sphere structure was not discerned with set of filters of Ex480/F520 or Ex565/F620. (**i**) Magnified OM image and fluorescence filter images of spheres on the SiN film indicated by a white arrow in (**b**). In all fluorescence filter images, ring-like structures were observed around the periphery of the spheres. (**j**) Magnified OM image and fluorescence filter images of the spheres attached to the slide glass indicated by a white arrow in (**f**). (**k**) Comparison of the line plots at the centre of the sphere image using the set of filters, Ex365/F420, on the SiN film and glass. The red, blue and green lines correspond to the RGB intensity values at the white dotted line position at the sphere centre, respectively (dotted line in (**i**) and (**j**) Ex365). The normalised RGB intensities of PS sphere on a SiN film show sharp peaks at the sphere ends (left plot). On the other hand, for the spheres on the slide glass, the blue line shows a dome shape, but the red and green intensity show no spherical structure but just noisy (right plot). Scale bars, 10 μm in (**a**) and 3 μm in (**j**).

Finally, we compared the fluorescence images of PS spheres attached to the SiN film and glass using a fluorescence microscope (Fig. 6). The Raman spectrum around the spheres showed a gradual shift to longer wavelengths than the incident laser at 532 nm (Figs. 1 and 2). Therefore, it is expected that the circumferential light that shifts to longer wavelengths than the excitation light can be observed using a conventional fluorescence microscope. The 3 μm PS spheres attached to the 50-nm thick SiN film were viewed under a fluorescence microscope, and the clear fluorescence images were observed with excitation light between 365 and 565 nm (Fig. 6a–d). In contrast, the spheres attached to the slide glass showed weak blue spherical autofluorescence with excitation light of 365 nm only (Fig. 6e,f). With excitation light of longer wavelengths such as 480 and 565 nm, the spherical shape completely disappeared (Fig. 6g,h). Since the spheres used in this experiment did not contain any fluorescent dye, only weak autofluorescence should normally be observed e.g. in the case of spheres attached to glass (Fig. 6f). Therefore, the fluorescence image of spheres on the SiN film certainly reflected light circulating around the sphere.

In the enlarged image of a single sphere on the SiN film, the ring-shaped fluorescence images were observed at any excitation wavelength (Fig. 6i). In contrast, in the case of the glass substrate, a weak spherical fluorescence image was observed only with 365 nm excitation light (Fig. 6j). Along the line going through the centre of the sphere at the SiN film with an excitation wavelength of 365, sharp peaks were seen at the boundary surfaces at both ends of the sphere at each detected intensity of red, green, and blue (Fig. 6k left). On the glass substrate, a dome-like shape showed the blue line plot, and green and red line plots showed just noise (Fig. 6k right). These results indicate that the excitation incident light on the spheres was gradually shifted to longer wavelengths while circulating around the outside surface of the sphere on the SiN film.

## Discussion

Here we show that anomalous Raman spectra are produced at the periphery of PS spheres on 50 nm thick SiN films (Figs. 1 and 2). This anomalous Raman spectrum shows a periodic amplitude change, which is expected to be due to interference (Fig. 5). However, there are several possible interpretations of the anomalous Raman spectra. The photons orbiting around the surface of the dielectric microspheres according to the proposal presented here have different characteristics from the normal WGMs orbiting inside the spheres. Normal WGM produces many sharp light intensity peaks and/or Raman spectrum peaks due to the resonance effect caused by the light circulating inside the fluorescence-doped spheres^8,21,23^. In our study, anomalous periodic Raman spectra were observed from PS microspheres without dye on SiN films, caused by interference of light orbiting outside the spheres. (Figs. 1 and 5). In general, light interference is produced by light reflected from interfaces and thin films; this has applications in interferometers and Fabry–Perot etalons.

There are several possible explanations for the generation of this anomalous Raman spectrum. One is the effect of resonance WGM on the contact surface between the WGM of the PS sphere and the SiN film. The film acts as a nanowaveguide channelling the light away from the sphere, with the efficiency having narrow peaks at the resonant WGM^32^. Therefore, the Raman signal is expected to show narrow dips at the correspondent spectral locations. Another effect that plays an important role in the waveform of the Raman spectrum is the Fano resonance linked to the internal triple mode of the sphere by WGM^36–38^. Fano resonance forms an asymmetrical peak in the spectrum, which may explain the shape of the anomalous Raman spectrum of the PS sphere. Due to this effect, it is possible that our results of the simple orbital light interference model shown here are not in complete accord with the Raman spectral waveform from the experiment. In addition to this, the effect of the photonic nanojet between the sphere and the SiN film may also be significant^34,35^. In the photonic nanojet, the focused light is directionally emitted from a very small region of the contact surface between the sphere and the SiN film, and this effect may form a large Raman amplitude. However, theoretical investigations and simulation analyses are needed to evaluate the influence of resonant WGM, Fano scattering and photonic nanojet on the anomalous Raman scattering spectra of dielectric spheres on a SiN film. In future we plan to perform simulation analysis using an optical model.

The difference in the ring light structure of PS spheres from confocal laser Raman microscopy (Figs. 1i, 3c, 4d and 5c) and fluorescence microscopy (Fig. 6i) can be attributed to two factors: one is due to polarization and the other to the observation system. In a confocal laser scanning microscope, the orbiting light changes depending on the irradiation position of the PS sphere due to the polarization characteristics of the laser, and the ring light structure of may be high in the equatorial region and low in the vertical direction. In addition, because the confocal laser Raman microscopy scans the laser, the laser irradiation time varies depending on whether the laser crosses the sphere centre or the upper and lower edges. Therefore, the ring structure may collapse. In fluorescence microscopy, the ring light shows a symmetric circular shape because the excitation light, which has no polarization properties, is irradiated over the entire sphere.

The periodic changes in the anomalous Raman scattering enhancement may be caused by the polarization of the excitation light. Therefore, for more detailed analysis, it is necessary to observe and compare the spheres on the SiN film while changing the polarization of the excitation light. Unfortunately, the Raman microscope used in this study cannot control the polarization of the laser. In the future, we plan to improve the instrument so that the polarization of the laser of the excitation light can be controlled, allowing us to perform more detailed analysis.

In conclusion, we report the strong enhancement of Ramana shift scattering in PS microspheres on SiN film. This enhancement occurs at the periphery of the sphere, and the same phenomenon can be observed by fluorescence microscopy. This is caused by the light circulating around the PS spheres as in the WGM. This circumferential light was observed most strongly when dielectric spheres of 3 μm diameter were placed on a 50-nm thick SiN film. The excitation light incident to the surface of PS spheres gradually shifts to a longer wavelength while orbiting outside of the sphere surface. Furthermore, interference among the circumferential lights produces a gradual periodic change in the Raman spectrum. These physical properties can be applied to high-sensitivity sensors, modulators, and quantum devices. Finally, our study uses ordinary materials such as PS spheres and SiN films as well as conventional measurement systems. In the near future, the system described here is expected to be widely utilized as a general-purpose platform for new optical devices.

## Materials and methods

### Sample preparation

The PS microspheres of 2–7 μm diameter suspended in aqueous buffer (Micromer, micromod Partikeltechnologie GmbH, Germany) were diluted three times with ultrapure water, vortexed for 10 s and sonicated for 1 min. The microsphere suspension (5 μL) was placed on a SiN film supported by a Si frame (4 × 4 mm^2^, 0.38-mm Si frame thick; Silson Ltd., UK) attached on the aluminium holder^29^, and 10 s later, the suspension liquid was absorbed by a piece of filter paper from the droplet. On a glass substrate, 10 μL of the microsphere suspension was placed on a slide glass (Micro slide glass S1111, Matunami Glass Ind. Ltd., Japan), and after absorbing the suspension liquid from the droplet by filter paper, the sample was dried for 5 min at a room temperature of 23 °C.

The Raman spectra of the PS microspheres were measured with air and water or oil underneath the SiN film. In the measurement condition of water or oil on the underneath the Si film, 5 μL ultrapure water or immersion oil (Type HF code 368, Refractive index 1.51–1.52, Cargille Labs. Inc., USA) was dropped into the centre hole of the aluminium holder, filled the underneath the Si film, and then the hole was covered with Kapton tape (tesa 51408, Tesa tape Inc., USA).

### Raman microscopy

The PS microspheres on a SiN film or slide glass were observed under a confocal laser Raman microscope using a 100× objective lens (Plan apo, Carl Zeiss, Oberkochen, Germany) and a 532-nm Nd-YAG laser (alpha300R, WITech, Ulm, Germany). Spectra were acquired with a Peltier-cooled charge-coupled device detector (DV401-BV, Andor, UK) with 600 gratings/mm (UHTS 300VIS, WITec, Germany). WITec suite (version 7.0, WITec, Germany) was used for data acquisition. For 2D Raman spectra, the laser intensity was 1.5–3 mW and the number of pixels in the XY axis was from 120 × 120 to 300 × 300. The pixel step width was 100 nm, and the measurement time for each pixel was set to 0.05 s. XZ axis scans were performed from 120 × 60 pixels, the step width set to X axis of 100 nm and Z axis of 200 nm. Raman spectral data were calculated using MATLAB R2020a (Math Works Inc., Natick, MA, USA) and plotted using Origin 2021J (Origin-Lab Co., Northampton, MA, USA).

### Calculation of Raman spectrum and 2D Raman image of PS spheres

The 2D Raman spectral data of the spheres were converted to the Matlab data file using WITec suite and were transferred to a personal computer (Intel Core i7, 3.2 GHz, Windows 10). The 2D Raman spectral images were calculated by Matlab R2020a with Image Processing Toolbox and Signal Processing Toolbox. The average Raman spectrum of the sphere centre was calculated from the pixels within a circle with a radius of 5 pixels from the sphere centre. For the spectrum of the sphere periphery, the Raman image of the PS peak of 1006 cm^−1^ was normalized after applying a 0.5σ Gaussian filter, and Raman spectra of the sphere periphery were averaged from pixels with normalized PS peak intensities between 0.2 and 0.5.

### Optical phase microscopy and fluorescence imaging

The microspheres of 3 μm in diameter attached to a slide glass (Matunami, Japan) and a SiN film were visualised at 400× magnification using an optical phase contrast microscope (AXIO Observer A1; Carl Zeiss, Oberkochen, Germany). Fluorescence images of the spheres were observed using fluorescence filters of the excitation/emission wavelength at 365/420 nm, 480/520 nm and 565/620 nm (Filter Set 02, 04 and 31, respectively, Carl Zeiss, Germany). The observed fluorescence images were normalised to an 8-bit scale by the maximum and minimum brightness intensity in each image. However, observation of spheres attached to glass slides using excitation light of 480 nm and 565 nm resulted in the disappearance of the sphere image. Therefore, the original images were shown in Ex480 and Ex565 of Fig. 6g,h,j to avoid noise enhancement due to normalization.

### Calculation of interference by orbiting light

The circumference of a sphere with diameter *D*_*s*_ is π*D*_*s*_. Interference due to light circulating around the spheres is assumed to be produced by light that has travelled around the sphere once and twice (*n* = 2). With the wavelength and sphere reflectance being *λ* and *η*_s_, the light amplitude *I*(*λ*) is given by the following equation.

2 $$I\left(\lambda \right)=\frac{1}{n}\sum \limits_{m=1}^{n}\mathrm{sin}\left(2\pi \frac{\pi {mD}_{s}{\eta }_{s}}{\lambda }\right) +1$$

The range of this equation is ± 1, which corresponds to the normalized light amplitude. Since the light amplitude does not take a negative value, we added 1 to make it greater than 0, and squared it to produce the light power (Eq. (1)). In the Raman spectrum of the PS sphere periphery, the anomalously enhanced Raman intensity is weak at short wavelengths, and the amplitude of the spectrum increases gradually with wavelength getting longer (Fig. 5). This wavelength-dependent optical amplification characteristic (amplitude coefficient) is denoted by *R*(*λ*) and is approximated by a characteristic which saturates exponentially as it shifts to longer wavelengths (Eq. (3)).

3 $$R\left(\lambda \right)=1-exp\left(-\frac{\lambda -{\lambda }_{i}}{\tau }\right)$$

Here *λ*_*i*_ is the wavelength of the incident laser, 532 nm. τ is the attenuation constant of the wavelength, set to 100 nm to fit the experimental data (τ = 100 nm). The calculation of *I*(*λ*), *R*(*λ*) and *P*(*λ*) for a 3 μm sphere with a wavelength between 532 and 640 nm is shown in Supplementary Fig. 6. The numerical calculations were performed using a PC (Windows10, 3.2 GHz, 8 core CPU: Core-i7) with Matlab R2020a.

## Supplementary Information

Supplementary Figures.

## Data Availability

All data generated or analysed during this study are presented in this paper or in the Supplementary Information. All the raw data files or spectra are available from the corresponding authors on reasonable request.
