# Supplementary material for: Raman scattering enhancement of dielectric microspheres on silicon nitride film
Source: Sci Rep. 2022 Mar 29;12:5346. doi: 10.1038/s41598-022-09315-5 (PMC8964696; doi:10.1038/s41598-022-09315-5)
Supplement: Supplementary file 1 — Supplementary Figures. [file 41598_2022_9315_MOESM1_ESM.pdf]

Supplementary information

Raman scattering enhancement of dielectric microspheres on silicon nitride film

Toshihiko Ogura[1]*

[1]Health and Medical Research Institute, National Institute of Advanced Industrial Science and Technology (AIST), Central 6, Higashi, Tsukuba, Ibaraki 305-8566, Japan

*Corresponding author: Toshihiko Ogura
Health and Medical Research Institute, National Institute of Advanced Industrial Science and Technology (AIST),
Higashi 1-1-1, Tsukuba, Ibaraki 305-8566, Japan
Tel.: +81-29-861-3408, Fax: +81-29-861-2677
E-mail: t-ogura@aist.go.jp

This PDF file includes:

Supplementary Figs 1 to 6

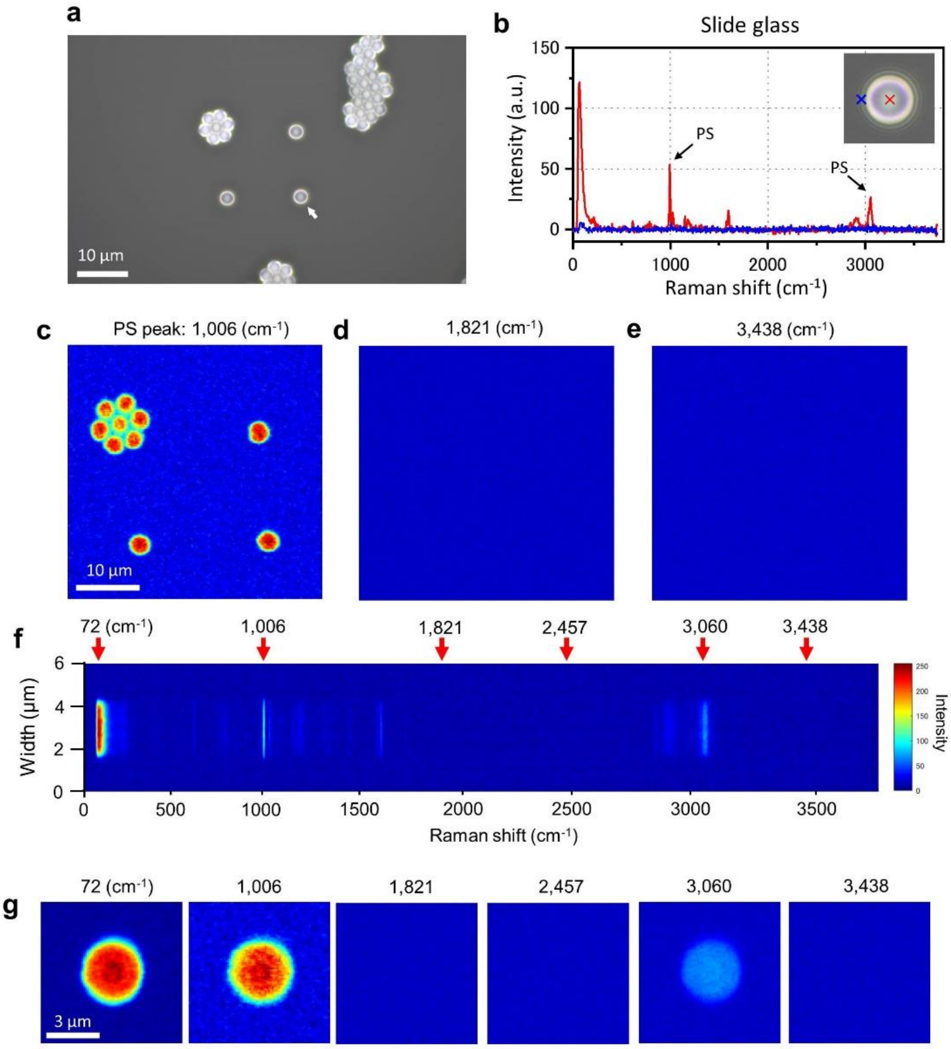

Supplementary Figure 1. Raman spectrum of PS microspheres on the slide glass. **a**, An OM image (×1,000) of 3 μm diameter PS spheres on the slide glass. **b**, Raman spectrum at the sphere centre (red) or at the left periphery (blue) on the glass indicated by a white arrow in **a**. **c**, Raman spectral peak image of PS (1,006 cm^{-1}). All the spheres in the scanned area were clearly observed. **d** and **e**, With the Raman images of 1,821 and 3,438 cm^{-1}, no spheres were discerned. **f**, A coloured Raman spectral map at the sphere centre indicated by a white arrow in **a**. The horizontal and vertical axes are the Raman spectral intensity and the width of the sphere centre, respectively. In this map, the light scattering peak (72 cm^{-1}) and the PS Raman peaks (1,006 and 3,060 cm^{-1}) are clearly observed. However, periodic anomalous Raman spectra were not observed. **g**, Raman images of a PS sphere indicated by a white arrow in **a**. The Raman images of spectral peaks (72, 1,006 and 3,060 cm^{-1}) show clear spherical structures. In the Raman images of 1,821, 2,457 and 3,438 cm^{-1}, the spherical structures were not observed. Scale bars, 10 μm in **a** and **c**, 3 μm in **g**.

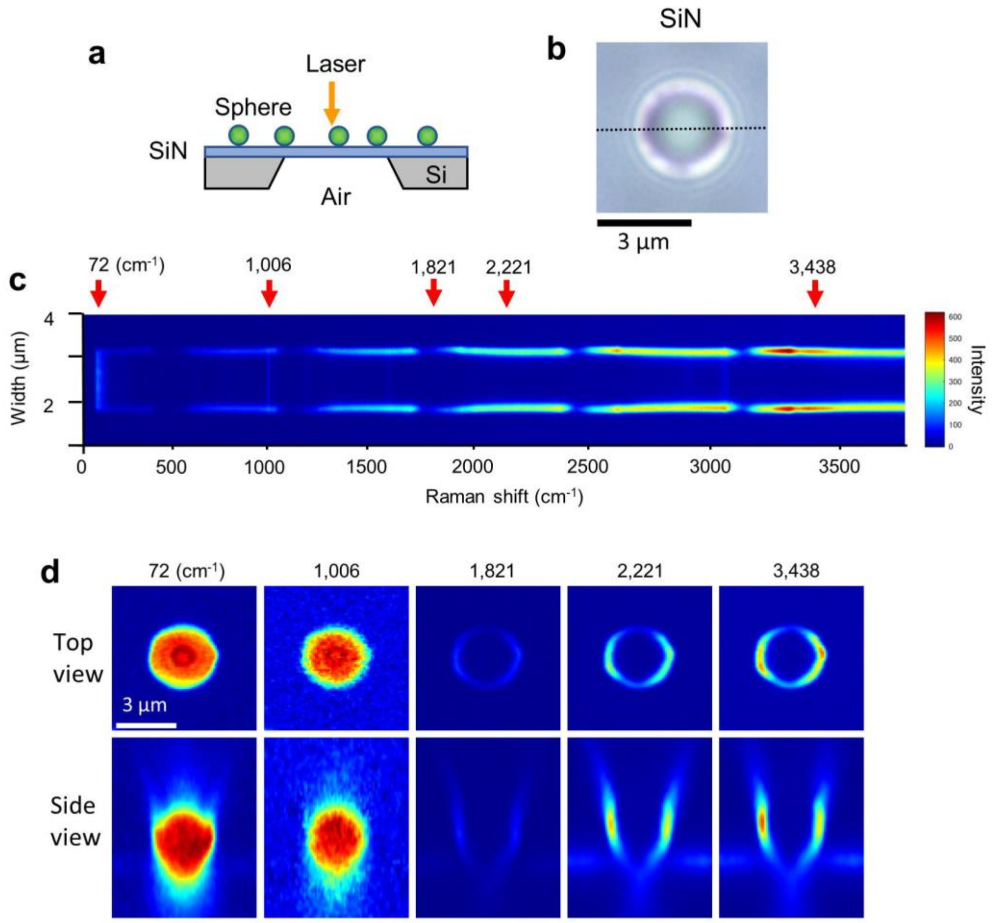

Supplementary Figure 2. Raman spectrum of PS microspheres on the SiN film.
a, Schematic of the measurement of Raman spectrum of 3 μm diameter PS spheres on a SiN film of 50 nm thickness. **b**, An OM image of a sphere on the SiN film. **c**, A coloured Raman spectral map at the sphere centre in **b**. Periodic high intensity Raman spectra are seen at both ends of the sphere. **d**, Top and side views of Raman images of PS sphere at the light scattering peak (72 cm^{-1}), the PS peak (1,006 cm^{-1}), the lower intensity of the anomalous Raman spectrum (1,821 cm^{-1}) and the higher intensity of anomalous Raman spectrum (2,221 and 3,438 cm^{-1}). In the Raman images of light scattering and PS peaks, the sphere shapes were clearly observed. In contrast, the peak images of the anomalous Raman spectrum showed a ring-like structure. Scale bars, 3 μm in **b** and **d**.

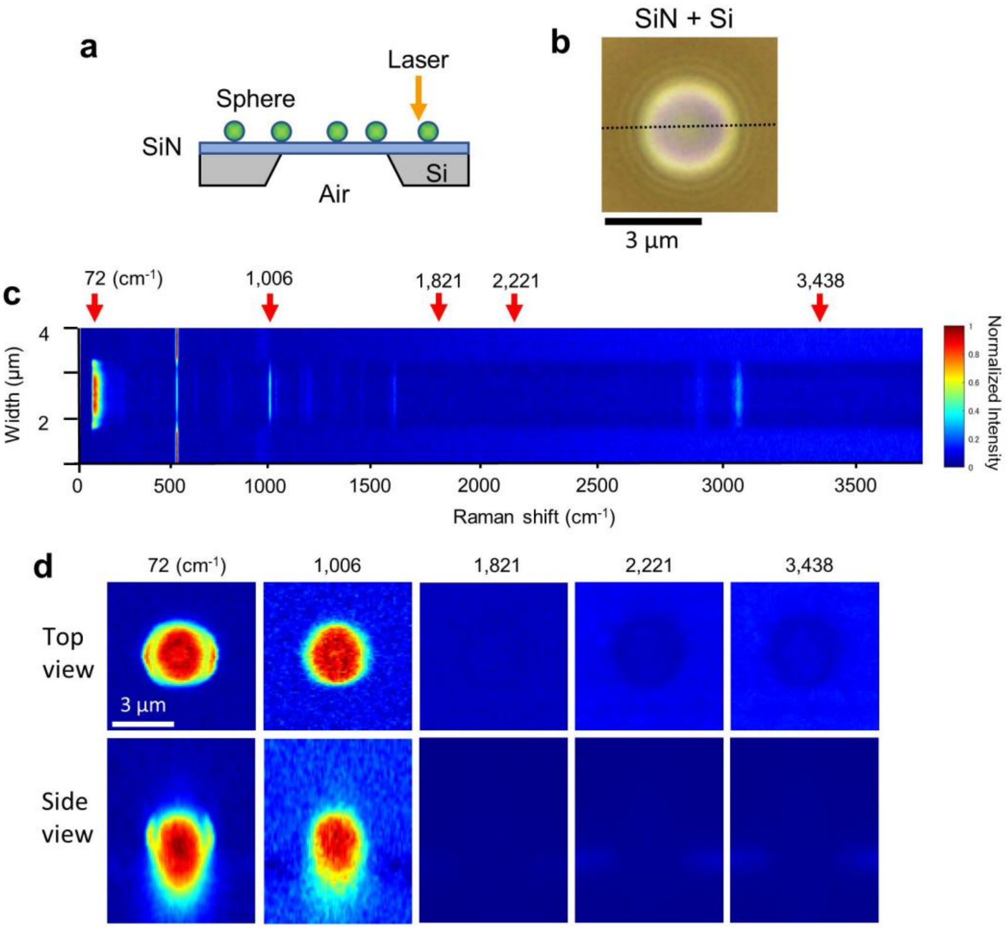

Supplementary Figure 3. Raman spectrum of PS microspheres on the SiN film contacted by a Si frame.

a, Schematic of the measurement of Raman spectrum of 3 μm diameter PS spheres on a SiN film of 50 nm thickness contacted by a Si frame. **b**, An OM image of a sphere on the SiN film contacted by Si frame. **c**, A coloured Raman spectral map at the sphere centre in **b**. Periodicity of high intensity of Raman spectrum, seen in Supplementary Fig. 2c, was not observed. **d**, Top and side views of Raman images of PS sphere on the SiN film contacted by Si frame. The ring-like structure of anomalous Raman spectrum image, seen in Supplementary Fig. 2d, was not observed (1,821, 2,221 and 3,438 cm^{-1}). Scale bars, 3 μm in **b** and **d**.

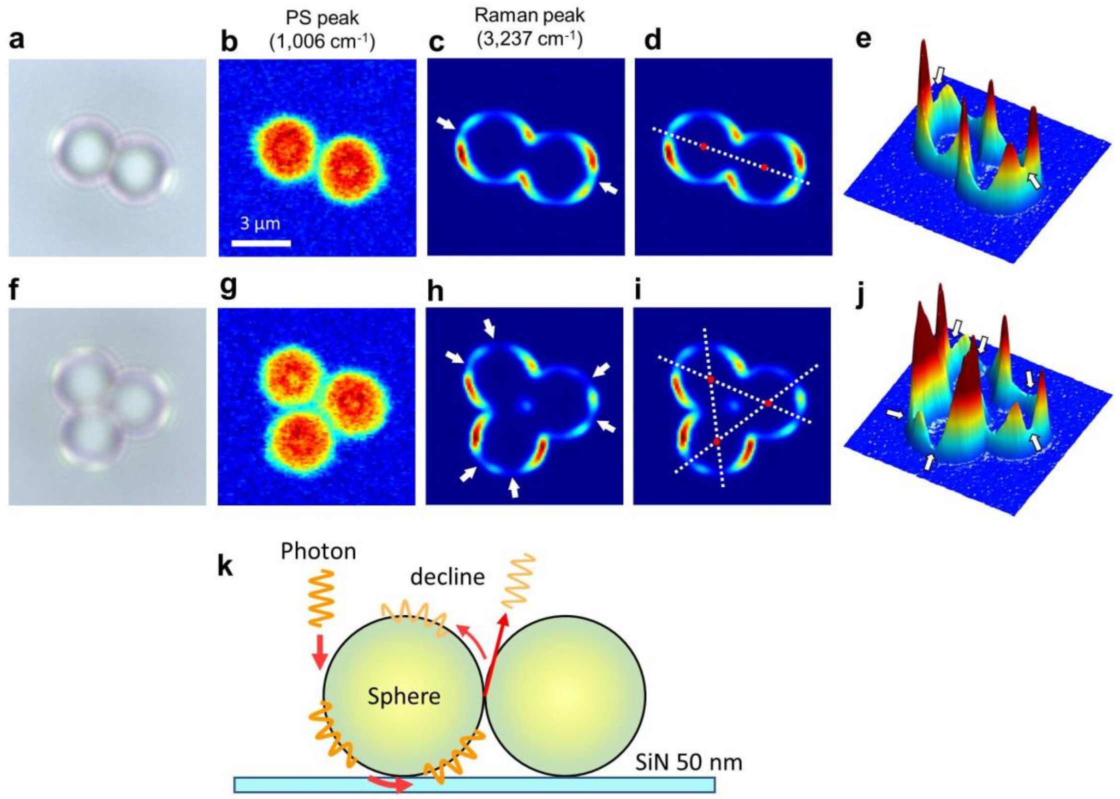

Supplementary Figure 4. Anomalous Raman spectra when multiple spheres are in close proximity on a SiN film.

a, An OM image of two spheres in close proximity on a SiN film. **b**, A Raman image of PS peak of 1,006 cm^{-1}. **c**, A Raman image of the peak of anomalous Raman spectrum (3,237 cm^{-1}). White arrows indicate localized attenuation points in the anomalous Raman spectrum in the sphere periphery. **d**, The attenuation points are located on a line (dashed white line) passing through the centre of each sphere (red points). **e**, Pseudo-3D colour maps of the anomalous Raman peaks on a SiN film. White arrows indicate localized attenuation points. **f–j**, Raman spectrum of three spheres in close proximity. **k**, Mechanism of localized attenuation points in anomalous Raman spectra. Scale bar, 3 µm in **b**.

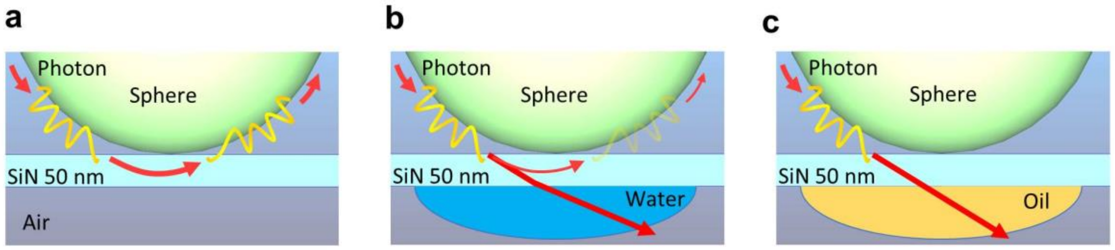

Supplementary Figure 5. Schematic diagram showing the substances underneath the SiN film affecting the orbiting light around spheres.

a, With air underneath the SiN film, the light circulating around the sphere passes through the SiN film. **b**, With water underneath the SiN film, the circumferential light around the sphere is attenuated. **c**, With immersion oil underneath the SiN film, the light circulating around the sphere disappears completely.

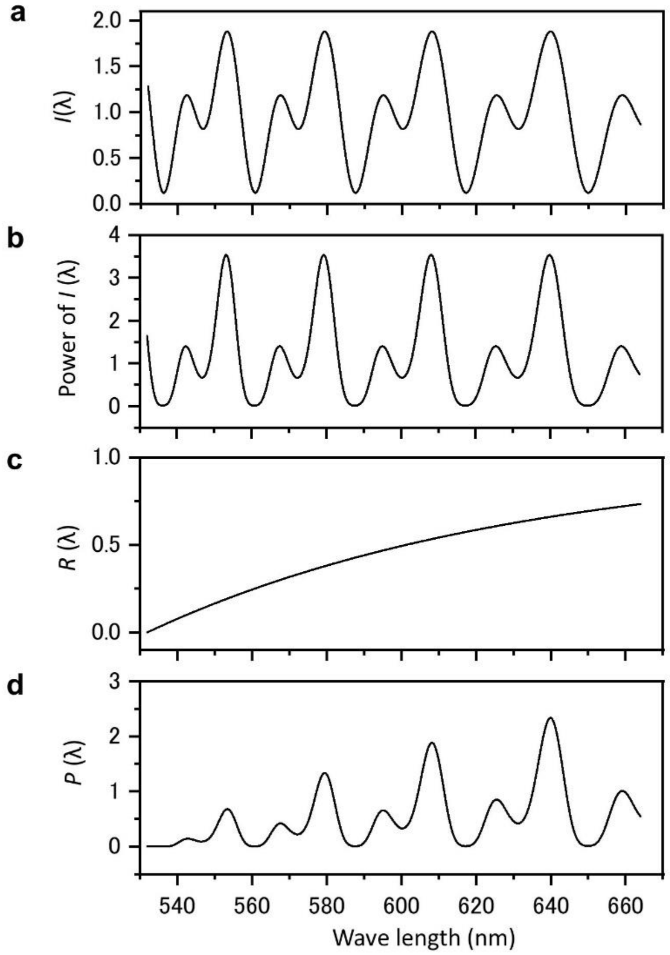

Supplementary Figure 6. Analytical plot of the mathematical model of light orbiting around a PS microsphere.

a, Plot of the interference light intensities $I(\lambda)$ according to equation (3), which has travelled twice and three times around the 3 μm diameter PS sphere. **b**, Plot of power of the interference light $I(\lambda)$. **c**, Plot of detection efficiency $R(\lambda)$ depending on wavelength according to (4). **d**, Intensity of the finally detected circumferential light $P(\lambda)$ according to equation (1).
